# Efficacy of high‐intensity interval training compared with moderate‐intensity continuous training on maximal aerobic potency in dogs: Trial protocol for a randomised controlled clinical study

**DOI:** 10.1002/vro2.4

**Published:** 2021-05-02

**Authors:** Sonia C. Orozco, María P. Arias, Pablo A. Carvajal, Jaime Gallo‐Villegas, Martha Olivera‐Angel

**Affiliations:** ^1^ Biogenesis Research Group Facultad de Ciencias Agrarias Universidad de Antioquia Medellín Antioquia Colombia; ^2^ INCACES group University CES Medellín Antioquia Colombia; ^3^ Servicio de Cardiología Hospital Veterinario Facultad de Ciencias Agrarias Universidad de Antioquia Medellín Antioquia Colombia; ^4^ Grinmade group Universidad de Antioquia Medellín Antioquia Colombia

## Abstract

**Background:**

High‐intensity interval training (HIIT) is a more efficient method to improve exercise capacity than moderate‐intensity continuous training (MICT) because of its greater physiological stimulus.

**Objective:**

The aim of this protocol is to evaluate the efficacy of HIIT on maximal aerobic potency in dogs as compared to MICT.

**Methods:**

This protocol is for a randomised, blinded controlled clinical trial, with three parallel groups for the purpose of demonstrating superiority. Thirty dogs aged between 12 and 84 months of both sexes and different breeds will be included. Dogs, before initiating and after finalising the training will perform an incremental exercise test on a treadmill to obtain maximal speed and lactate threshold; resting parameters of heart and respiratory rate, left ventricle chamber and systolic function will be measured. Dogs assigned to each intervention will endure a 42‐min session of HIIT or MICT during 12 weeks. HIIT comprises four intervals of 4 min each at a load of 85%, alternating with a 4‐min resting period. MICT group will have a continuous load of 60%. The control group will remain in a cage. An intention‐to‐treat statistical analysis will be implemented. Analysis of covariance will be used to estimate the effect of HIIT compared with MICT training on maximal aerobic potency, aerobic resistance, systolic function at rest, left ventricle chamber measurements and indexes, respiratory rate and HR at rest.

**Conclusion:**

Significant time and effort are invested into training sports/working dogs, which could benefit from improving physical capacity by means of the HIIT methodology.

## INTRODUCTION

Dogs are used in sports and working roles and many exercises on a regular basis with their owners. Therefore, numerous canines engage in routine physical activity.^1^ In order to achieve a top athletic performance, they need to reach an adequate level of task fulfillment.^2^

Aerobic potency is the maximum energy production by oxidative routes.^3^ Improved aerobic potency in dogs enhances cardiovascular and respiratory capacity that helps increase work capacity and sports performance.^4,5^ It may also cause a lower respiratory rate with improving thermoregulatory performance that contributes to a higher sniffing rate^6^, ^7^ and olfactory acuity.^8^ Increased maximal aerobic potency lessened the cardiac output^9^ in senescent dogs, and physical conditioning was achieved in dogs with induced acute myocardial infarction.^10^

High‐intensity interval training (HIIT) provides superior or equal benefits to moderate‐intensity continuous training (MICT) for maximizing health outcomes in humans but with less time commitment.^11,^, ^12^ In humans, its main goal is to improve the maximum amount of oxygen used during exercise or its maximum aerobic potency.^3^ HIIT is characterised by alternating periods of high intensity with light recovery exercise or no exercise periods.^13^ High‐volume HIIT protocols in humans are defined as those that accumulate 15 min or more during high‐intensity intervals, with all other HIIT protocols being defined as low volume.^14^ HIIT enables a greater physiological stimulus and adaptation as compared to MICT for maximal aerobic potency in humans,^15^ horses^16^ and dogs.^17^ High‐volume protocols have shown greater efficacy in improving cardiorespiratory fitness as compared with MICT in persons, and low‐volume HIIT protocols have shown similar but not superior improvements compared with MICT.^4^

Training methods can determine different types of physiological improvements, where the use of a sole or a combination of these is used to improve health and athletic parameters in humans.^13^ MICT as a training method has been extensively used and researched in dogs. HIIT constitutes a training alternative which may possibly generate a potential benefit in physically active dogs. Most of the dog sports and working tasks require bouts of moderate to intense physical activity that alternate with a less active or resting phase.^18,^, ^19^ HIIT training is a more specific training for the type of activities that dogs carry out. So, it is biologically feasible that HIIT's near‐maximal effort may provide physical fitness improvement, as to a higher velocity developed, more frequent bouts, less recovery time between them or a delay on the onset of fatigue in the field.

There are no guidelines of HIIT in dogs; so, there is no clear definition of time, number of intervals, recovery time or classification of low and high volume.^17^ There is hardly any research in this area.^20^ Because of the nature and trainability of dogs, this method is feasible and could be used to enhance physical fitness. The physiological adaptations to this type of training in dogs need to be documented. The efficacy of HIIT as compared to MICT on maximal aerobic potency in palatability testing of dogs needs to be established. The purpose of this study is to compare the efficacy of two aerobic training programs on the maximal aerobic potency in non‐exercising dogs. The authors aim is to test the hypothesis that HIIT training is superior as compared to MICT in increasing the maximal aerobic potency in dogs.

## MATERIALS AND METHODS

### Study design

A randomised controlled clinical trial that will use the minimization method for the allocation to ensure that each treatment group will be balanced with respect to the predefined factors of age, size and sex, as well as for the number in each group.^21,^, ^22^ The professionals who will evaluate the outcomes will be blinded to the treatment group of each dog. Three parallel treatment groups will be formed for the purpose of demonstrating superiority of HIIT compared to MICT^23^ based on the primary outcome of Maximal aerobic potency.

**FIGURE 1:**
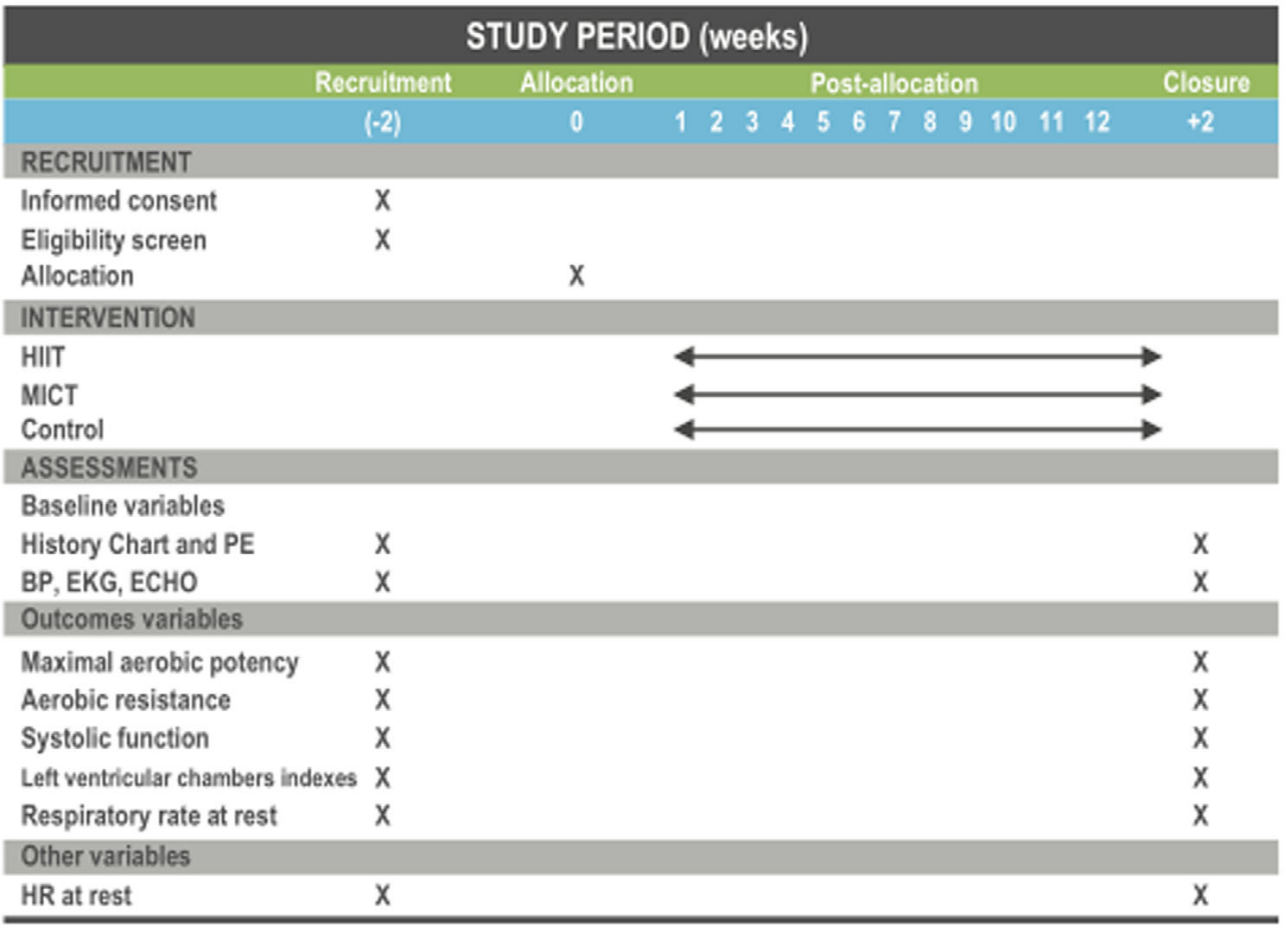
General design of the study: recruitment, intervention and assessment. Once informed consent has been signed by the administrator of the kennel, a complete physical examination (PE), blood pressure measurement (BP), echocardiogram (ECHO), ECG and blood work will be performed. After defining the inclusion/exclusion criteria, recruited dogs will undergo an incremental exercise test (IET) and an ECHO. The dogs will be assigned to a HIIT, MICT or Control group intervention. Afterward, all the dogs will undergo a reevaluation IET and ECHO. The recruitment and closure periods are projected as 2 weeks. The intervention will be 12 weeks.

Dogs aged between 12 and 84 months of both sexes and different breeds will be included and will undergo a physical examination, blood pressure measurement, echocardiogram, ECG and routine blood work. Maximal speed, lactate threshold (LT), resting parameters of heart rate (HR), respiratory rate, left ventricle chamber dimensions and systolic function will be measured both before and after 12 weeks of a treadmill exercise program. The design was based on the SPIRIT protocol, guidelines for the elaboration of interventional trials protocols,^24^ adjusted to a canine population shown in Figure 1.

### Animals and setting

The study shall be carried out in a closed population kennel of a commercial dog food company (Table 1), in Envigado, Colombia, located at 1.726 m above the sea level with year‐round temperatures between 17°C and 28°C and a humidity of 75−85%. The kennel has the same handling, water/food, housing, vaccination and deworming conditions for all the dogs. The dogs were spayed and neutered. Their body condition score will be monitored every 2 weeks to maintain them at a 4−5/9.^25^

**TABLE 1:** Characteristics of the 49 dogs of the kennel population

	**Small**		**Medium**		**Large**		**Total**	
**Baseline characteristics**	*n*	%	*n*	%	*n*	%	*n*	%
**Individuals**	25	51.0	12	24.5	12	24.5	49	100.0
**Sex**								
Females	9	36.0	8	66.7	5	41.7	22	44.9
Males	16	64.0	4	33.3	7	58.3	27	55.1
**Age (months)**								
Mean ± SD	34.3 ± 28.5		35.5 ± 28.6		32.7 ± 19.2		34.2 ± 26.1	
**Weight (kg)**								
Mean ± SD	7.5 ± 3.1		18.2 ± 2.1		32.7 ± 6.2		16.3 ± 11.1	

### Eligibility criteria

Healthy sedentary dogs aged between 12 and 84 months will be included. Healthy status shall be defined as 'in physiological range' of physical parameters, blood work and ancillary diagnostic aid parameters. Dogs with cardiovascular, neuromuscular, or orthopedic conditions, pregnant or lactating bitches, body condition score over 7, ^25^ or any other clinical abnormalities that could impair exercise performance will be excluded from the study.

### Sample size

A total sample size of 30 dogs (HIIT, *n* = 10; MICT, *n* = 10; C, *n* = 10) is calculated, by using STATA software *t*‐test iteration, a two‐sided test, assuming a difference between HIIT and MICT of 0.57 m/s in maximal velocity achieved, a standard deviation of 0.35 m/s,^26^ a confidence level of 95%, an 80% power and a 1:1:1 ratio and 20% of potential withdrawals.

### Intervention

The training program will be conducted in the kennel in a well‐ventilated room, controlled by the kennel veterinarian who is independent of the research. Cooling fans will be used during incremental exercise tests (IETs) and training sessions. The dogs will wear a safety harness. Food will be withdrawn 2 h before each exercise session, and only water will be available. IETs and training sessions will be held in the morning between 7 AM and 11 AM, where room temperature range from 17°C to 20°C. The dogs will be stretched, and a lubricant will be applied to the paws at the end of each session.

The training sessions will be conducted four times per week during the first 4 weeks, the remaining sessions will be conducted three times per week, with a progressive 1.25% of increase in loads for HIIT every week and for MICT, a progressive 1.25% of increase in loads every 2 weeks.

To verify that a trained state will be obtained, a greater than or equal to 15% reduction in HR at rest will be required.^27^, ^28^ The dogs assigned to each intervention (*n* = 10) will endure a 42‐min session of HIIT or MICT. The velocity at LT (VLT) will correspond to the treadmill speed at the LT.^26^ The HIIT comprises four intervals at a load of 85−95 per cent of the VLT of the IET, with no inclination, alternating with a 4‐min no exercise period. The MICT group consists a load of 60–65% of VLT, with no inclination. The control group will remain in a cage for the equivalent time of the training session.

**FIGURE 2:**
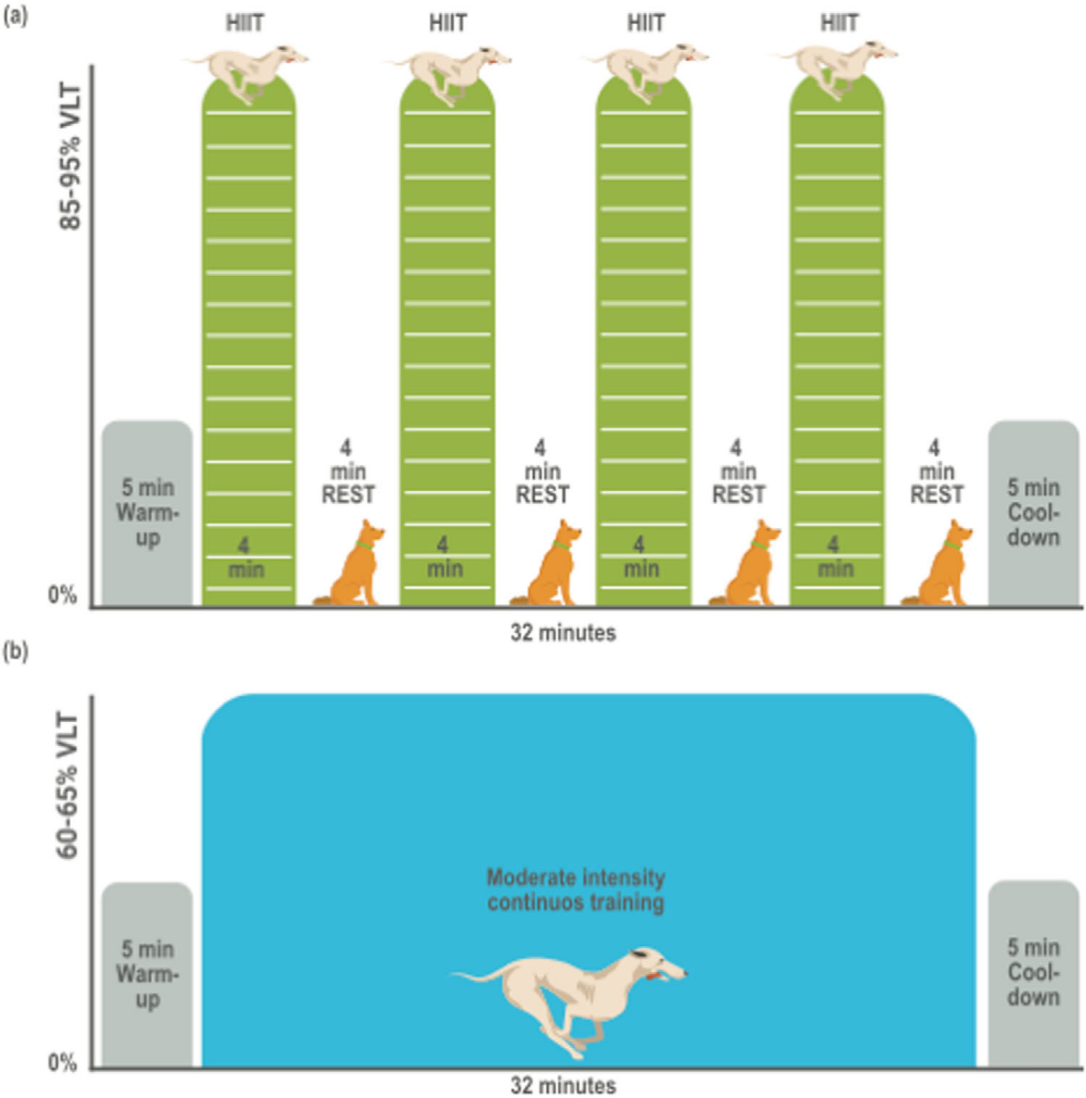
Training protocols. Both protocols will have a 5‐min warm‐up and cool‐down period. (a) High intensity interval training protocol (HIIT) will have four intervals at a high intensity at an 85−95% of VLT (velocity at the lactate threshold) interspersed by four resting periods. (b) Moderate‐intensity continuous training protocol will have a 32‐min exercise period at a 60−65% of VLT

HIIT program: The HIIT session will include a warm‐up and cool‐down period of 5 min each at 4.8 km/h, followed by four intervals of 4 min each interspersed by a 4‐min no exercise period, for a total duration of 42 min. The intervals will be at 85% of VLT of the IET, with a progressive 1.25% of increase in loads every week up to 95% of VLT (Figure 2a).

MICT program: The MICT session will include a warm‐up and cool‐down period of 5 min each at 4.8 km/h, followed by 32 min at an intensity of 60% of VLT of the IET, for a total duration of 42 min. A progressive 1.25% of increase in loads up to 65% of VLT will be made every 2 weeks (Figure 2).

### Strategy for training adherence

Adherence will be established from the percentage of the total volume of exercise performed in relation to the total exercise programmed. An adherence greater than or equal to 80% will be considered for statistical per‐protocol analysis. All dogs will be included in the intention‐to‐treat analysis, independent of their percentage of adherence. Dogs will receive treats during and after each session, verbal and physical positive reinforcement. No dog will be forced; this process will be made as joyful as possible.

### Outcome measurements

All outcome measurements will be performed 48−72 h before the intervention and 48−72 h after the final last training session. The measurements will be carried out during the morning, with a minimum of 2 h after feeding.

**FIGURE 3:**
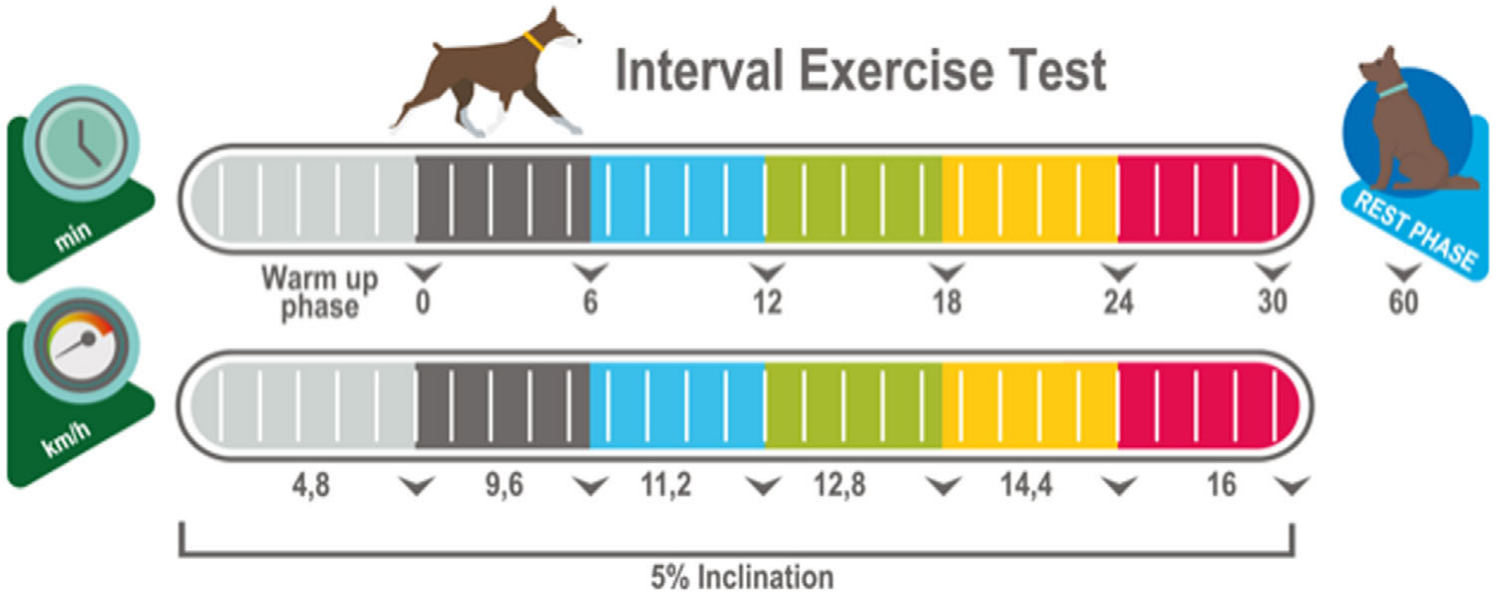
Incremental exercise test (IET). An 8‐min warm‐up phase at 4.8 km/h with a 5% of inclination precedes the IET. The IET has five stages of 6 min each at increasing speeds of 9.6, 11.2, 12.8, 14.4 and 16 km/h with a 5% of inclination. A short pause between stages will be implemented to obtain blood samples to determine the lactate levels. A lactate sample will be drawn every 10 min after finalizing the IET during the 30‐min resting period

Maximal aerobic potency (primary outcome). An IET on a treadmill will be executed before and after completing the intervention. The maximal velocity (Vmax) that each dog develops during each IET will be compared. The IET will be executed as described,^4^, ^29^, ^30^ an additional 8‐min warm‐up at 4.8 km/h at a 5% inclination will be implemented.The IET will have a maximal duration of 38 min, consisting of five stages of 6 min each, starting at 9.6 km/h, incrementing 1.6 km/h every stage up to 16 km/h (Figure 3).

Aerobic resistance (secondary outcome). Blood LT will be measured during the IET at the end of every stage as described,^4^, ^29^, ^30^ before and after completing the intervention. A lactate sample will be drawn every 10 min after finalising the IET during the 30‐min resting period. LT will be defined as the point on the plasma lactate–velocity curve at which there is an increase in lactate concentration.^26^, ^31^ Blood lactate levels will be determined with a handheld lactate meter (Lactate Plus Meter, NOVA, Waltham, USA) validated for dogs.^32,^, ^33,^, ^34^

Systolic function at rest (secondary outcome). A veterinary cardiologist will perform a routine echocardiogram; no sedatives will be used. A mean of three measurements will be calculated for each echocardiographic variable. Left ventricular ejection fraction, using the Simpson's method of discs,^35,^, ^36,^, ^37^ and shortening fraction will be calculated from M‐mode images as current recommendations.^36,^, ^38,^, ^39^ Peak systolic velocities will be recorded from the septal and lateral mitral annular sites as current guidelines by using tissue Doppler imaging.^35,^, ^38,^, ^40^

Left ventricle chamber measurements and indexes (secondary outcome). Left ventricular mass and relative wall thicknesses will be calculated based on M‐mode echocardiographic measurements according to current recommendations.^41^, ^–^, ^43^ Left ventricular mass and relative wall thicknesses will be calculated as described by Devereux.^37,^, ^41,^, ^44^

Respiratory rate and HR at rest (secondary outcome). Both measurements will be taken while the dog is alone resting in his pen, without any contact with the personnel. The respiratory rate will be determined by a 20‐min video footage taken by a camera on a tripod outside the pen. The HR will be measured with a HR monitor during 20 min (Polar Electro Oy, Model H10, Kempele, Finlandia); the receptor will be placed outside the pen.

### Recruitment

The administrator of the kennel signed an informed consent to review and to recruit the eligible individuals. The history charts of the 49 active palatability dog testers will be assessed to define eligibility as each one fulfills inclusion and exclusion criteria. A complete physical examination will be performed with emphasis on cardiovascular, neuromuscular and orthopedic systems. Blood pressure, ECG and echocardiogram will be measured according to standard recommendations.^35,^, ^45,^, ^46^

### Allocation of the interventions, implementation and blinding

The SICOR Clinical Trial Coordinating Center, which is external to the researchers, will generate the randomization allocation sequence at a 1:1:1 ratio (HIIT, *n* = 10; MICT, *n* = 10; C, *n* = 10), with the minimization method, with the predefined subject variables of age (12−120 months), size (small: less than 15 kg; medium: greater than 15 kg and less than 25 kg; large: greater than 25 kg), and sexes (male or female). A base probability of 0.7 will be used by the 'bias coin' method and variance as a measure to define the difference between each group, for the addition of the next dog.^21^, ^22^ The MinimPy v0.3 open source will be used.

The SICOR coordinator will send to the lead investigator the assignment of each intervention group through e‐mail. The outcome measurements will be performed by professionals who will be blinded to the allocation to each group. One of the investigators will compile the data of the training sessions on tailored digital files on an online backup service, with a password that will be accessible to the lead investigator. Each dog will have a code to ensure blinding of his training regimen for the statistical analysis process.

### Statistical analysis

An exploratory analysis will be carried out; the Shapiro–Wilks test will be used for the analysis of normality. The mean and the standard deviation, the median, the interquartile range and percentage will be used for the variables. A multiple imputation method (five imputations) will be used for the lost data based on age, sex, size and baseline HR. An intention‐to‐treat analysis will be implemented. Individuals having an adherence greater than or equal to 80% by pre‐protocol analysis will be accomplished.

Analysis of covariance will be used to estimate the effect of HIIT compared with MICT training on maximal aerobic potency as the primary outcome, aerobic resistance, systolic function at rest, left ventricle chamber measurements and indexes, and respiratory rate and HR at rest at the end of the training period. Homogeneity of variance assumption will be assessed with Levene's F‐test. This test will also be used to assess the linearity and homogeneity of the regression slopes. If the assumptions of normal distribution are not met, a logarithmic transformation of the variables or a square transformation will be performed; the Box–Cox procedure will also be performed. A re‐randomization procedure will be carried out using permutations in order to infer.^21^ The level of significance will be set at *p* < 0.05 (two‐sided) for all comparisons. STATA v14.0 and IBM SPSS Statistics v21.0 will be used.

### Data collection, monitoring committee and auditing

Digital files will be created to record the information of baseline characteristics, each training session and the proposed outcomes. The lead investigator and the kennel veterinarian will have access to the database. No monitoring committee will be constituted because of the brief term of the trial and the minimal risk of the intervention. No interim analysis will be scheduled. Every week the lead investigator will review the archives to ensure thorough information of the clinical trial and access to the final dataset. The database will be secured on an online backup service.

### Adverse events

The proposed training regimens are considered safe. The reported frequency of adverse events related to these training types is very low, mainly musculoskeletal. The possible adverse musculoskeletal, heat stroke and exercise‐induced collapse, or other clinical abnormalities developed during the training will be monitored and registered in each history chart by the kennel veterinarian, who has the authority to remove an individual or stop a training session. Staff will be trained to identify clinical signs of heat stress and heat exhaustion, so dogs will be promptly removed before they develop heat stroke.

### Ethical principles

This trial is based on the Law 84 of 1989 and Law 1774 of 2016 of Colombia, which regulate against animal suffering and pain inflicting, caused directly or indirectly. No electrical shock or coercing can be performed.

### Protocol amendments, ancillary and post‐trial care

If a dog exhibits lameness because of the training program, it will be rested for a maximum time of 2 weeks; if it continues to exhibit lameness, it will be removed from the intervention. The kennel veterinarian, who is independent of the research, will determine treatments, diagnostic protocols and removal of a dog if it develops any health alteration that could impair exercise performance. The kennel will cover all the medical expenses that may arise.

### Consent, confidentiality and dissemination policy

The administrator of the kennel signed the informed consent for the participation of all the kennel dogs (supplemental material). All the information will be kept in confidence until the training process is over. The results will be declared to the kennel administrator once all the intervention is completed. The researchers will define a strategy to report the outcomes as academic original articles and posters in scientific events. It is intended to publish the full protocol of this trial.

## DISCUSSION

Traditionally, dog training regimens have been focused on MICT protocols. HIIT is a possible alternative that improves physical fitness in dogs.^17,^, ^20^ Most of the dog sports and working tasks demand bouts of moderate to intense physical activity that alternate with a less active or resting phase^47^ that is similar to HIIT. Lee et al stated HIIT was suitable for dogs, and it might prevent cardiovascular and metabolic diseases.^20^ However, neither physiological adaptations nor health benefits to this type of training in dogs are extensively documented.

Ready and Morgan investigated active sled dogs with HIIT protocol. They observed reduction of the lactate after 12 weeks of HIIT at the same maximal velocity of the final IET compared to the dogs in the continuous protocol. The authors concluded that HIIT produced a physiological response that improved the aerobic potency, implied by the decrease of the lactate at the final stage of the IET as compared to the initial stage, even though the dogs were previously in a trained state.^17^

In Lee's study, two dogs were subjected to a HIIT and two to a MICT; HRs were recorded during each session. The HRs of the HIIT dogs were seen to be positively correlated to the intensity increments, but the HRs of the dogs in the MICT increased when the treadmill had an inclination over 6%. In this report, even though no prior or final IET was performed to compare the HRs of both protocols, and the training lasted only 4 weeks, the average HR maximum of HIIT was 199 beats per minute (bpm) compared with 141 bpm in the MICT protocol. HIIT elicited a greater physiological stimulus to the cardiovascular system that was reflected in the higher HRs, which could enhance aerobic potency, even in this short period of time.^20^ Both studies proved that HIIT was found superior in increasing aerobic potency as compared to MICT.

In three studies, dogs with various stages of myxomatous mitral valve disease, after 8 weeks^48,^, ^49^ and after 24 weeks in another study of continuous training,^50^ significant physical capacity,^49,^, ^50^ quality‐of‐life, functional class improvement^50^ and augmented aerobic capacity were determined.^48^ Quality‐of‐life was determined by improvement in dogs’ behavior, alertness, physical activities and skeletal muscle tone.^50^ Myxomatous mitral valve disease is the most frequent heart disease in dogs, where low‐volume HIIT may provide a more efficient cardiovascular adaptation, because of its accumulated time in HIIT compared with MICT protocols. Physiological responses and adaptations to HIIT have not been evaluated in dogs with heart disease or any other disease.

As there are no guidelines for HIIT in dogs, so there is no clear definition for time, number of intervals, recovery time or classification of low and high volume. Research is scarce in this area.^17,^, ^20^ Low‐ and high‐volume HIIT definition can be extrapolated from human guidelines; however, it needs to be validated. A non‐active resting phase is apparently best; a slow pace decreases dogs’ concentration; whereas, a complete resting phase allows the dog to fulfil the next interval. Swanson and others described that dogs get easily distracted at lower treadmill speed.^20^, ^51^ At this time, these are only observations. It is necessary to wait for the results of this clinical trial, where several more questions will probably arise.

In humans, exercise prevents at least 25 medical conditions^12^ and morbi‐mortality associated with chronic diseases.^15^ High‐volume HIIT protocols have shown greater efficacy in improving cardiorespiratory fitness compared to MICT in humans.^14^ Higher exercise intensities may be superior to moderate intensity for maximizing health outcomes.^12^ Because of these attributes, extensive research in humans has been focused on the subsequent adaptations which are variable and depend on the intensity, duration and number of intervals implemented.^14,^, ^52^ The improvement of aerobic potency in physically active dogs is relevant because of its health benefits to enhance cardiovascular and respiratory capacity, which in turn may contribute to increased work capacity and sports performance.^4,^, ^5,^, ^26^ It also allows maintenance of the cardiac output throughout life span.^53^ Lee et al found reduced cholesterol levels, no change in hematological parameters, slight decrease in glucose and triglycerides, increased creatine kinase that returned to the normal after the HIIT program, which imply this to be an alternative method to prevent metabolic diseases and improve body composition in overweight or obese.

A possible limitation of this study is that training protocols were matched by workload based on the perceived exertion scale (PES) for dogs.^51^ Therefore, the MICT protocol has 32 min with an intensity of 2 PES, and HIIT has 16 min with an intensity of 4 PES, both having a workload of 64. Measurement of oxygen consumption and energy expenditure in dogs requires expensive equipment. Gerth and others had developed a formula to predict energy expenditure based on HR in large dogs (28 ± 3 kg) that were exercised at 60–65% of their maximal aerobic capacity^54^; this formula cannot be applied to dogs under and over that weight or above that percentage of intensity.

The sample size of this research is based on the results of 18 beagles that endured 8 weeks of MICT training,^26^ because of the lack of research on dogs with HIIT training. This could be a potential limitation; the genetic variability of the breeds of this study is a confounding variable, which will be balanced with the minimization method to each intervention group. Also, as the sample size for this intervention is small, it might have a low chance of detecting an important effect on the outcomes.

Dogs have muscle fiber types 1, 1–2A, 2A, 2A‐2X, 2X. 2A muscle fibers are most abundant in the trunk and limb muscles; they have an aerobic oxidative metabolism that contribute to the resistance to fatigue in endurance exercise.^55^ The neuromuscular system recruits faster motor units during intense exercise to generate muscle tension reaching maximum effort.^56^ Because of these features, it is reasonable that aerobic oxidative capacity can be improved by the near‐maximal effort, in particular, when starting from low levels of physiological adaptation to exercise.

Extensive research has been made on the changes of dogs’ heart function and left ventricular dimensions because of MICT training.^10,^, ^28,^, ^57^, ^–^, ^59^ HIIT's effects on heart function was described by Ready^17^ and Lee.^20^ It is not known whether the intensity defined for the intervals, the time of each interval, the accumulated time of the intervals and duration of this protocol could cause changes on the left ventricular dimensions and systolic function.

Significant time, effort and finances are invested into the raising and training of sports/working dogs.^1^ To achieve and maintain top athletic performance, these dogs need to reach an adequate level of task fulfillment.^2^ Adequate training is essential for successful maximal physical conditioning; it could avoid exercise‐related problems that can result in critical downtime and compromise its career.^2,^, ^29,^, ^57,^, ^60^ Dogs that are routinely trained for sports and working reasons could benefit from the effective and efficient way of improving physical capacity with the aid of HIIT methodology.

## AUTHOR CONTRIBUTIONS

Planning of the study design was conducted by María P. Arias, Pablo A. Carvajal, Martha Olivera‐Angel, Jaime Gallo‐Villegas and Sonia C. Orozco. Jaime Gallo‐Villegas calculated the sample size and statistical analysis plan. Review of the final draft of the manuscript was performed by all authors. Figures were designed and provided by Sonia C. Orozco.

## FUNDING INFORMATION

This research was supported by Colciencias grant (National Doctorates 757/2018), Solla and Nova Biomedical (Waltham, MA, USA). The funders did not participate in data collection and analysis, article writing or submission.

## ETHICS APPROVAL

Ethical approval was obtained from the committee of animal experimentation of the Universidad de Antioquia (120‐09‐10‐2018).
